# Supplementary material for: Haptic human–human interaction does not improve individual visuomotor adaptation
Source: Sci Rep. 2020 Nov 16;10:19902. doi: 10.1038/s41598-020-76706-x (PMC7670433; doi:10.1038/s41598-020-76706-x)
Supplement: Supplementary file 1 — Supplementary Figures. [file 41598_2020_76706_MOESM1_ESM.pdf]

Haptic human-human interaction does not improve individual visuomotor adaptation

Supplementary Information

Niek Beckers[1,2,*], **Edwin van Asseldonk**[1], **and Herman van der Kooij**[1,3]

[1]Department of Biomechanical Engineering, University of Twente, Enschede, The Netherlands
[2]Cognitive Robotics, Delft University of Technology, Delft, The Netherlands
[3]Department of Biomechanical Engineering, Delft University of Technology, Delft, The Netherlands
[*]Correspondence should be addressed to N.B. (niekbeckers@gmail.com)

Supplementary Figures

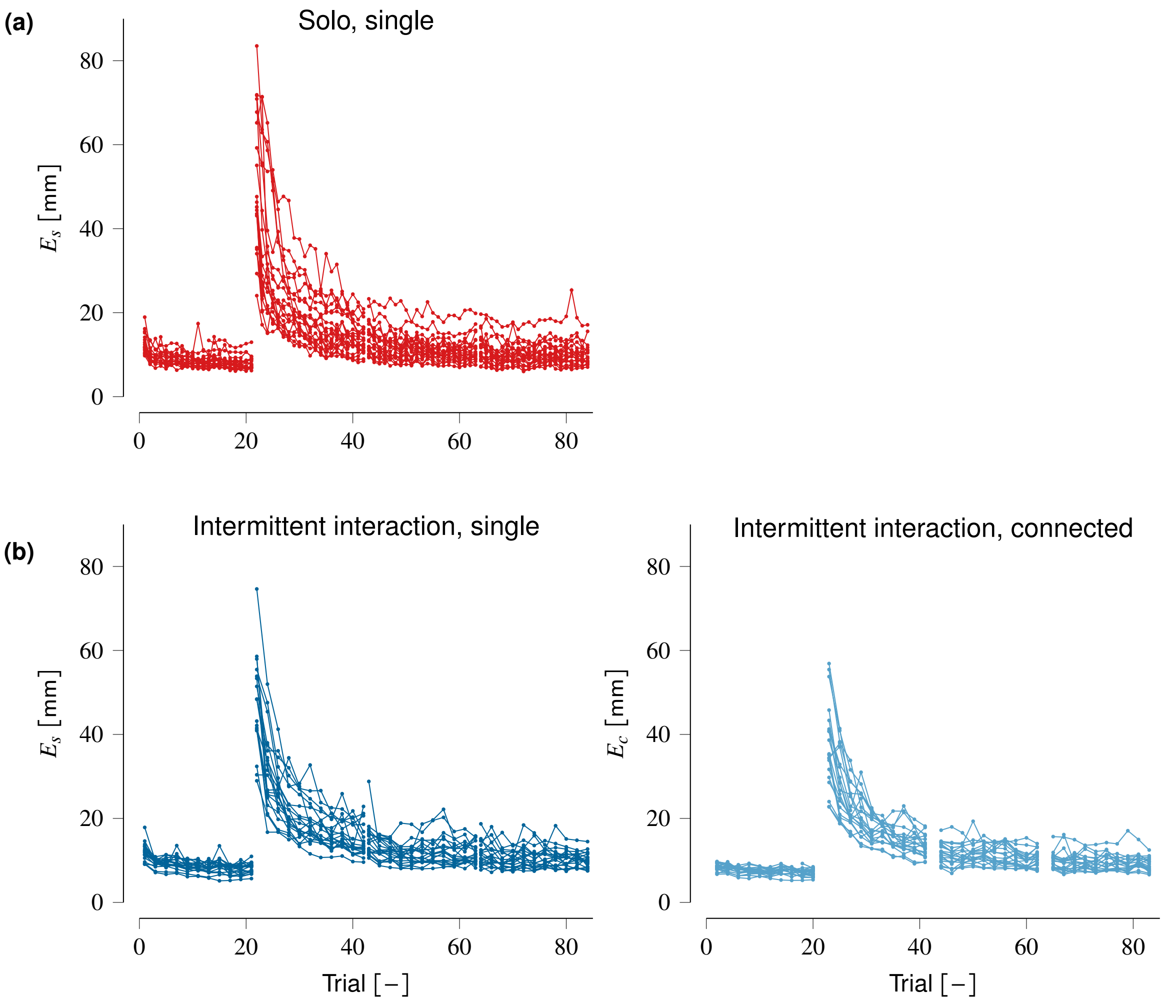

Supplementary Figure S1. Single trial tracking error (E_s) and connected trial tracking error (E_c) curves for every participant. **(a)** Solo group, **(b)** intermittent interaction group, **(c)** stiff interaction group and **(d)** Continuous interaction group (participant 11 is also included).

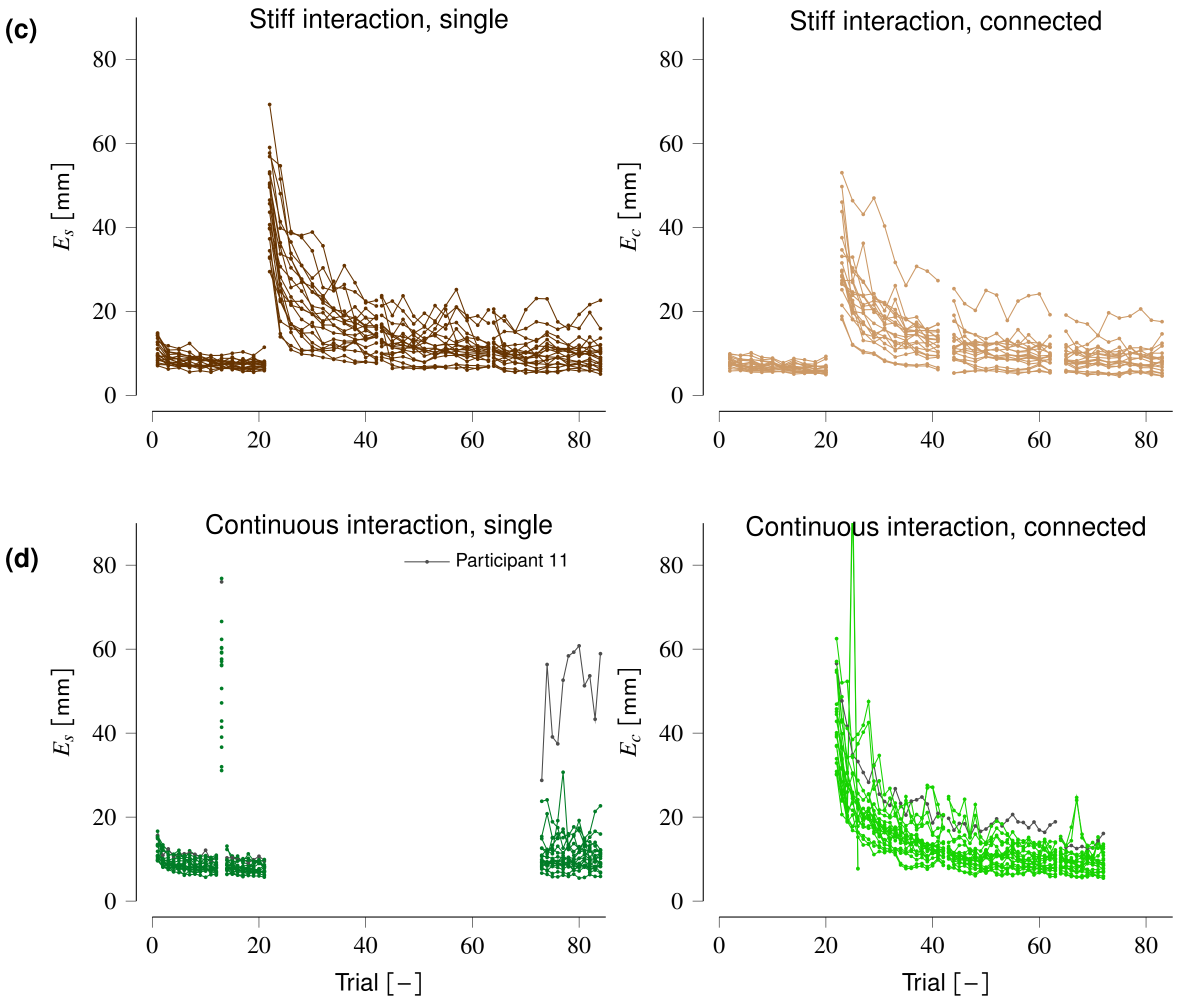

Supplementary Figure S1. Single trial tracking error (E_s) and connected trial tracking error (E_c) curves for every participant. **(a)** Solo group, **(b)** intermittent interaction group, **(c)** stiff interaction group and **(d)** Continuous interaction group (participant 11 is also included).

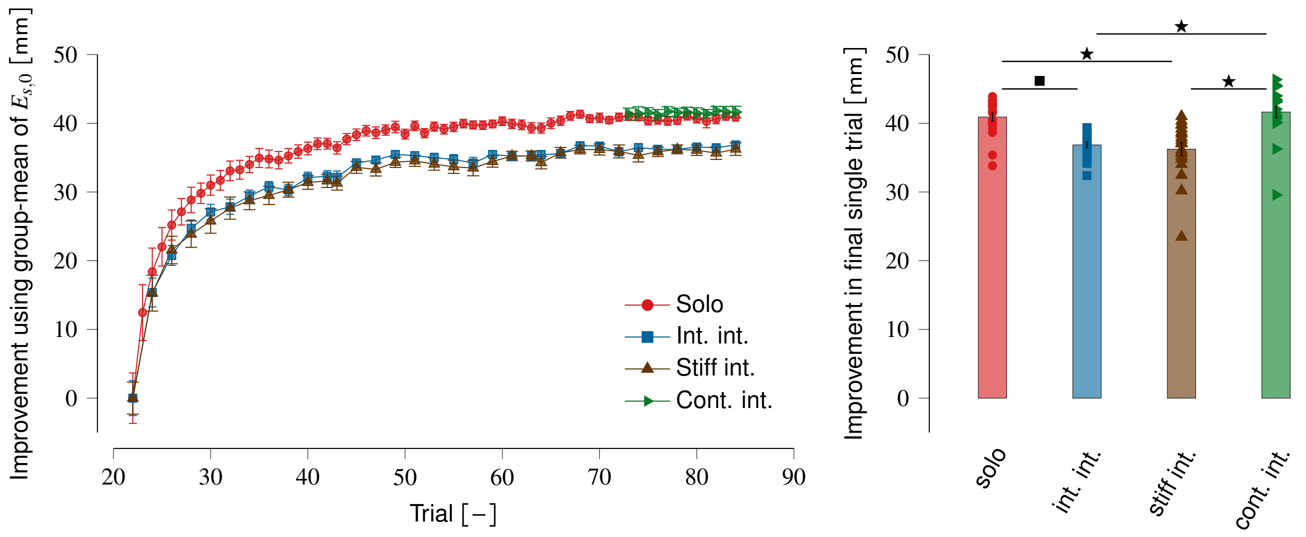

Supplementary Figure S2. Improvement based on the group-mean of the initial tracking error $E_{s,0}$ (i.e. $(I_s)^{gm} = E_s - (\overline{E}_{s,0})^{gm}$). This improvement calculation is the same approach as used by Ganesh et al.[1]. We found a significant difference in improvement across groups in the final single trial (right figure) using a linear mixed-effect model with group as dependent variable and pair as random variable (group factor: $\chi^2(3) = 24.47$, $p < 2 \cdot 10^-5$). Post-hoc tests using a Tukey correction showed significant differences between groups, which are indicated in the figure (■: $p < 0.005$, ★: $p < 0.001$). Interestingly, intermittent interaction leads to *less* improvement compared to the solo group using this improvement calculation approach. The solo and cont. int. group showed similar improvement. This would mean that *intermittent interaction* would impede individual motor improvement, which is opposite to Ganesh et al.[1] conclusion. A stiffer interaction results in significantly lower improvement compared to the solo and cont. int. groups.

Supplementary Methods

Target signal design

The target signal is defined as a quasi-random sum-of-sine signals which are designed following human-in-the-loop tracking signal design guidelines[2]. The target signal movement $f_{x,y}(t)$ in x and y was generated using

$$f_{x,y}(t) = \sum_{k=1}^{4} A_{k_{x,y}} \sin\left(\omega_{k_{x,y}} t + \varphi_{k_{x,y}}\right), \tag{1}$$

where $A_{k_{x,y}}$ is the amplitude, $\omega_{k_{x,y}}$ is the frequency and $\varphi_{k_{x,y}}$ is the phase of sine k. Both sum-of-sines in x and y consist of four sines, each with a different amplitude, frequency and phase which are listed in Supplementary Table S1.

The frequencies spanned a frequency bandwidth of $0.943\,\mathrm{rad\,s^{-1}}$ to $3.142\,\mathrm{rad\,s^{-1}}$, similar to Ganesh et al.[1]. The frequencies were multiples $n_{k_{x,y}}$ of the measurement time base frequency $\omega_m = 2\pi/T_m = 0.3142\,\mathrm{rad\,s^{-1}}$ (the measurement time for each trial was $T_m = 20\,\mathrm{s}$). The frequency multiples and frequencies are given in Supplementary Table S1.

We used a second-order low-pass filter to determine the amplitudes:

$$|A(j\omega)| = \left|\left(\frac{1+T_1 j\omega}{1+T_2 j\omega}\right)^2\right|, \tag{2}$$

where $T_1 = 0.1\,\mathrm{s}$ and $T_2 = 0.8\,\mathrm{s}$. The amplitude set in x and y were both scaled to a variance of $12\,\mathrm{cm}^2$. The reduced amplitudes at the higher frequencies yields a target movement that is not overly difficult, but still results in quasi-random movement.

Phases were selected from a large set of randomly generated phases such that the resulting time traces of the target signal had a minimum crest factor (no excessive peaks in the time traces)[2].

Supplementary Table S1. Target signal sum-of-sine parameter values.

k	$n_x\,[-]$	$\omega_{k,x}\,[\mathrm{rad\,s^{-1}}]$	$A_{k,x}\,[\mathrm{cm}]$	$\varphi_{k,x}\,[\mathrm{rad}]$	$n_{k,y}\,[-]$	$\omega_{k,y}\,[\mathrm{rad\,s^{-1}}]$	$A_{k,y}\,[\mathrm{cm}]$	$\varphi_{k,y}\,[\mathrm{rad}]$
1	3	0.94	2.87	−7.77	4	1.26	2.71	−0.71
2	4	1.26	2.71	−8.53	5	1.57	2.53	−3.45
3	6	1.89	2.35	−4.36	7	2.20	2.16	3.92
4	9	2.83	1.80	−3.79	10	3.142	1.64	4.93

References

1. Ganesh, G., Takagi, A., Osu, R., Yoshioka, T. & Kawato, M. Two is better than one: Physical interactions improve motor performance in humans. *Sci. Reports* **4** (2014).
2. Damveld, H. J., Beerens, G. C., van Paassen, M. M. & Mulder, M. Design of Forcing Functions for the Identification of Human Control Behavior. *J. Guid. Control. Dyn.* **33**, 1064–1081 (2010).
